# A Rare Case of Colonic Gastrointestinal Stromal Tumor

**DOI:** 10.7759/cureus.60383

**Published:** 2024-05-15

**Authors:** Aravindan K, Jayaganesh P

**Affiliations:** 1 Pathology, Saveetha Medical College and Hospital, Chennai, IND

**Keywords:** immunohistochemistry, risk stratification, colon gist, gist, gastrointestinal stromal tumor

## Abstract

Gastrointestinal stromal tumors (GISTs) are a type of mesenchymal tumor of the gastrointestinal tract that originate anywhere in the gastrointestinal tract, with the colon and appendix being the least recorded sites of occurrence. The following case report is that of a colonic GIST in a 53-year-old male and its histologic type. Included are notes on the recent additions and updates in the risk stratification of GISTs occurring in unusual sites with the relevant immunohistochemistry.

## Introduction

Mesenchymal tumors form the majority of primary non-epithelial neoplasms of the gastrointestinal tract. Broadly called gastrointestinal stromal tumor (GIST), it can arise from any part of the gastrointestinal tract, such as the stomach, omentum, mesentery, etc. Less than 5% of GISTs occur in the colorectum [1] (usually the rectum). They tend to occur in adults, are mostly histologically malignant, and are mainly treated using chemotherapy followed by a complete resection of the mass. Gastrointestinal stromal tumors were earlier called leiomyomas or leiomyosarcomas. They comprise the most common tumors in the gastrointestinal tract, which are of mesenchymal origin. However, they make up only a small percentage of all gastrointestinal tumors.

The incidence of GISTs is around 4500 cases annually. They are thought to arise from the interstitial cells of Cajal [2], which are mesodermal-derived cells in the wall of the gastrointestinal tract that control gut motility. Gastrointestinal stromal tumors therefore have the potential to occur throughout the gastrointestinal tract but most commonly occur in the stomach (60%), followed by the jejunum and ileum (30%), duodenum (5%), and colon and rectum (5%), and rarely the esophagus and appendix. The characterization and diagnostic criteria of GISTs have progressed greatly in the past 10 years through immunohistochemical staining, with the discovery that a majority of GISTs stain for the cell surface markers CD-117 (95%), PDGFRA (93%), and DOG1 (95%) [3]. Much progress has also been made in the identification of prognostic features of GISTs, with their larger size (>5 cm) and higher mitotic rate (>5 mitoses per high power field) indicating a worse prognosis [4].

## Case presentation

**Figure 1:**
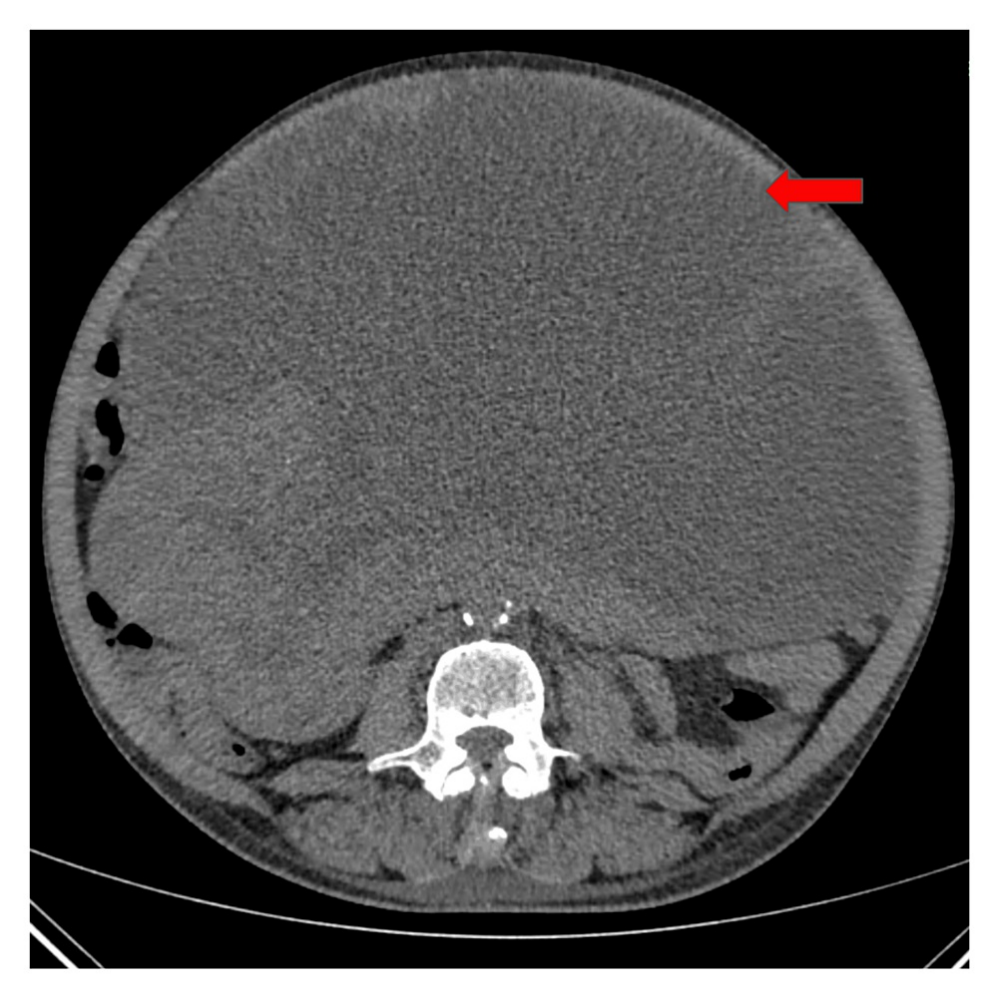
The CECT of the abdomen shows a large pelvic-abdominal, heterogenous, mixed-solid cystic space-occupying lesion (red arrow). CECT: Contrast-enhanced computerized tomography

This case is that of a 53-year-old male with a history of mass abdomen who had undergone previous chemotherapy. He presented with complaints of abdominal mass with distension, breathlessness, and loss of weight. On contrast-enhanced computerized tomography (CECT) abdomen, a large pelvic-abdominal heterogenous, mixed-solid cystic space-occupying lesion (Figure 1) was seen extending from the epigastric region cranially to the suprapubic region caudally. The entire mass, along with a portion of the adjacent transverse colon up to the splenic flexure, was excised and sent to the pathology department for histopathological examination.

**Figure 2:**
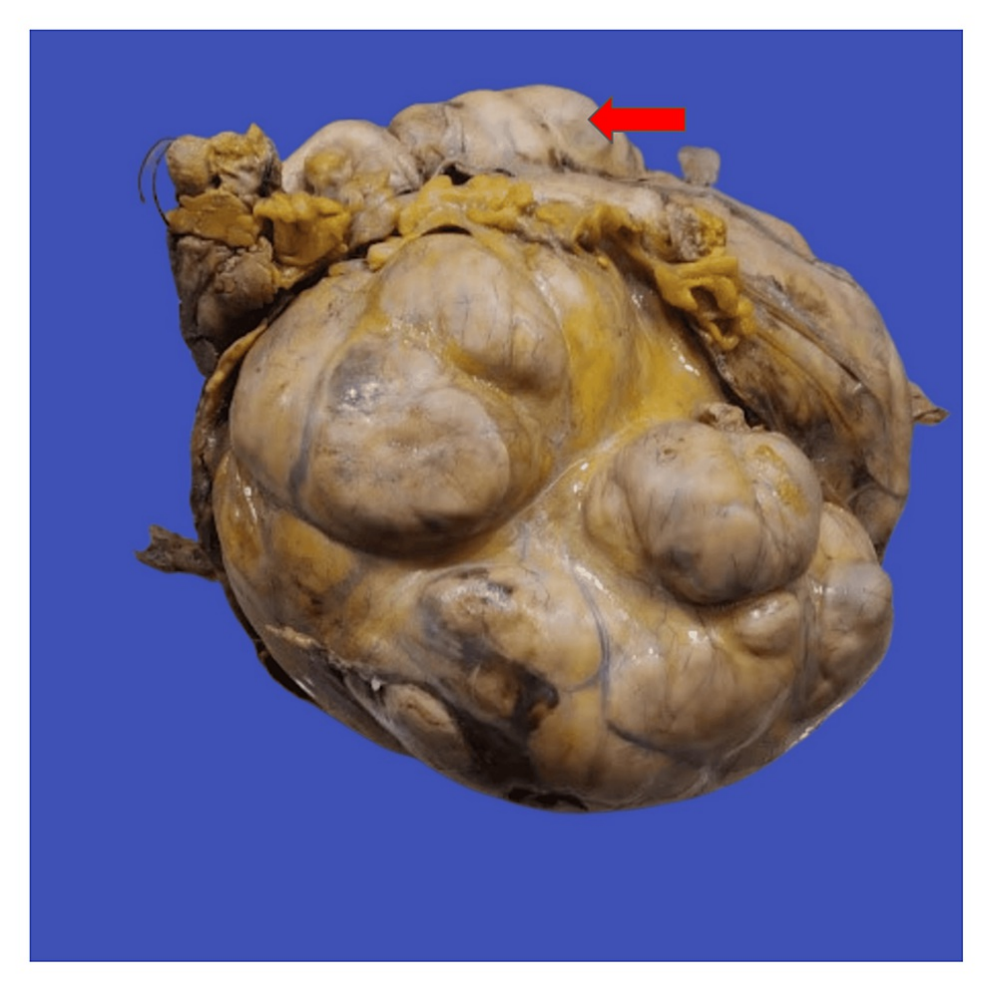
Encapsulated, lobulated mass showing no capsular breach with the attached large intestine segment (red arrow)

**Figure 3:**
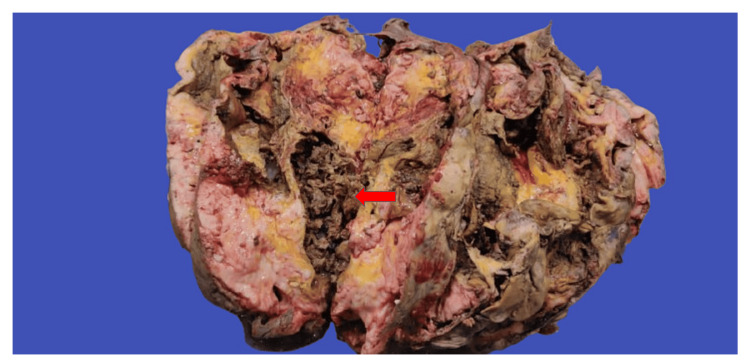
Cut surface of the mass showing areas of necrosis (red arrow)

Upon macroscopic examination, an encapsulated mass measuring 28x21x18 cm (Figure 2) was seen with the attached segment of the large intestine measuring 15.4 cm. The external surface was lobulated with no capsular breech, and the cut surface appeared fleshy and gray-white with areas of necrosis (Figure 3).

**Figure 4:**
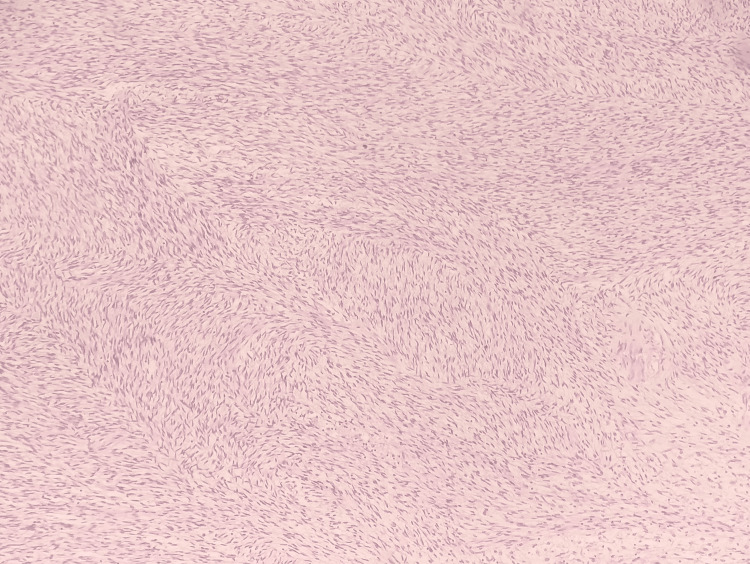
Hematoxylin and eosin stain (20x objective) shows spindle cells arranged in fascicles

**Figure 5:**
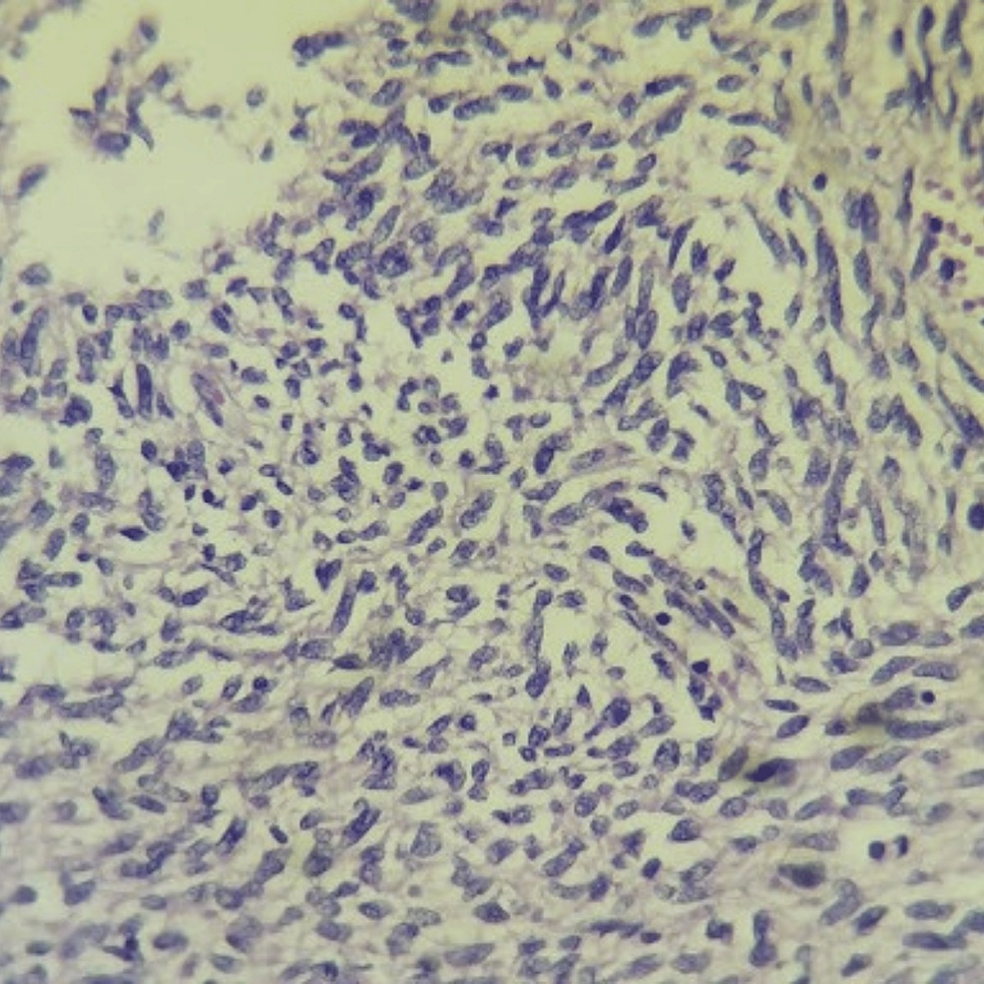
Hematoxylin and eosin stain (40x objective) shows spindle cells having a moderate amount of eosinophilic cytoplasm

Microscopic examination showed a neoplasm arising from the submucosa of the colon, composed of spindle cells arranged in fascicles (Figure 4), with a moderate amount of eosinophilic cytoplasm (Figure 5) and a brisk mitotic rate of greater than five per 5 mm^2^. Hence, a provisional diagnosis of GIST was given, and further immunohistochemical staining was advised to confirm the diagnosis. No regional lymph nodes were submitted separately for examination.

**Figure 6:**
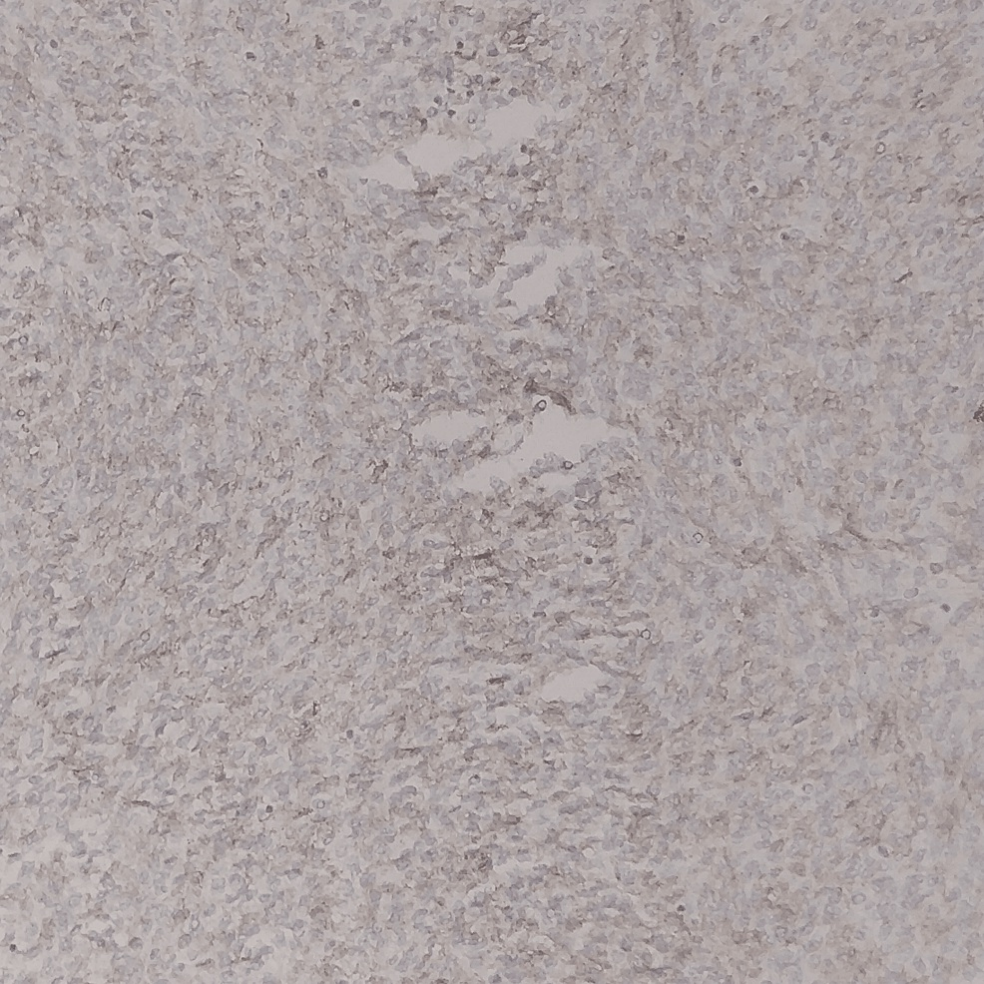
Immunohistochemistry (40x objective) shows tumor-cell positivity for DOG1

An immunohistochemical stain for DOG1 (discovered on GIST) was found to be positive (Figure 6). This confirmed the diagnosis of GIST, and based on the risk stratification criteria of site, size, and mitotic activity, which are discussed below, this case was classified under the high-risk category. The patient was started on chemotherapy with imatinib, and on a recent follow-up, he showed no signs of recurrence.

## Discussion

Gastrointestinal stromal tumors can arise from any part of the gastrointestinal tract, though the stomach and small bowel are the most common sites. A GIST in the esophagus, colon, or appendix is extremely uncommon, and cases in the rectum are rare. Though no longer used, the older terminology for GIST was gastrointestinal autonomic nerve tumor (GANT) [1] and leiomyoblastoma.

A GIST is usually submucosal and can range from sizes as small as 1 cm to >50 cm. They can grow either exophytically [5] or endophytically. Though smaller-sized GISTS can be asymptomatic unless found out incidentally during routine screening procedures, the most common clinical manifestation of larger-sized GISTS is a large abdominal mass and its associated pressure effects. The clinical symptoms can vary from intestinal obstruction to GI bleeds.

Grossly, the GISTs are well-encapsulated tumors with a red, fleshy-cut surface that can show areas of hemorrhage and necrosis [6], especially in very large tumors due to degeneration, as seen in this current case. Microscopically, they are composed of spindle cells [7] arranged in fascicles and can show occasional palisading. An important finding in this spindle cell GIST is the presence of paranuclear halos, which is a formalin fixation artifact. Another type is the epithelioid GIST, which is more aggressive. On the genetic aspect, almost all GISTs harbor mutations either in the cKIT [8] pathway or the PDGFRA [9] pathway, the identification of which can lead to targeted therapy [10]. Some studies have shown that GISTs with PDGFRA mutations are usually of the epithelioid type [11]. 

Immunohistochemically, GISTs are positive for CD34, which is non-specific [12]. The specific markers for GIST are CD117, or cKIT, and DOG1, also known as Ano1. These markers show diffuse positivity. Among the aforementioned two markers, DOG1 is the most specific marker, as some cases of cKIT and CD 34-negative GISTs also showed positivity for DOG1 [13,14], meaning DOG1 positivity is independent of cKIT or PDGFRA mutation status.

The risk stratification of GIST is based on the site, the size, and the mitotic activity seen in the tumor. The mitotic activity is measured as per 50 high power fields or 5 mm^2^. The preferred method for mitotic count is per 5 mm^2^, as the area counted can vary based on the lens present in the microscopes [15]. In 2002, Fletcher et al. proposed one of the earliest criteria for risk stratification (Table 1) [16].

**Table 1:** Risk stratification criteria HPF: High power field Reproduced from *Diagnosis of gastrointestinal stromal tumors: A consensus approach *by Fletcher et al. [16]. This table has been used with permission from Elsevier Inc.

Risk	Tumor size	Mitotic count
Very low risk	<2 cm	<5 / 50 HPF
Low risk	2-5 cm	<5 / 50 HPF
Intermediate risk	<5 cm	6-10 / 50 HPF
	5-10 cm	<5 / 50 HPF
High risk	>5 cm	>5 / 50 HPF
	>10 cm	Any mitotic rate

The updated risk stratification based on the size, site, and mitotic rate for GISTs arising in unusual locations is given in Table 2 [17].

**Table 2:** Updated risk stratification for GISTs occurring in unusual sites The risk of disease progression is classified as none, moderate, or high-risk of disease progression. Reproduced from *Gastrointestinal stromal tumors (GISTs) arising in uncommon locations: Clinicopathologic features and risk assessment of esophageal, colonic, and appendiceal GISTs *by Hu et al. [17]. This table has been used with permission from Elsevier Inc. GIST: Gastrointestinal stromal tumor

Mitotic rate	Size of the tumor (cm)	Esophagus	Colon	Rectum	Appendix
≤ 5 per 5 mm2	≤2 cm	None	None	None	None
	>2 to ≤5 cm	None	None	Low	
	>5 to ≤10 cm	Moderate	Moderate	High	Insufficient data
	>10 cm				Insufficient data
> 5 per 5 mm2	≤2 cm	Insufficient data	None	High	Insufficient data
	>2 to ≤5 cm	High	Moderate	High	Insufficient data
	>5 to ≤10 cm	Moderate	High	High	Insufficient data
	>10 cm				Insufficient data

As for the reported case, based on the aforementioned risk stratification criteria per size (more than 10 cm), site (colon), and mitotic rate (greater than five per 5 mm^2^), it was classified under high risk. Therefore, the patient was started on imatinib chemotherapy. The recent follow-up showed no recurrence.

## Conclusions

Gastrointestinal stromal tumors can involve any part of the gastrointestinal tract, with the stomach being the most common site. Large intestine GISTs are rarer and mainly involve the rectum, with colonic GISTs being even rarer. They tend to occur in adults, are mostly histologically malignant, and are mainly treated using chemotherapy followed by the complete resection of the mass. In conclusion, this case report highlights the diagnostic challenges and therapeutic considerations encountered in the management of GISTs. Through a multidisciplinary approach involving thorough clinical evaluation, advanced imaging modalities, histopathological examination, and molecular profiling, accurate diagnosis and tailored treatment strategies have been achieved. In spite of being a common mesenchymal tumor of the GIT, not enough data is available for GISTs occurring in even rarer locations, such as the appendix. Hence, further research and collaboration are essential to enhancing our understanding of this entity.
